# Meigs’ syndrome with elevated CA 125: case report

**DOI:** 10.1590/S1516-31802003000500007

**Published:** 2003-09-01

**Authors:** Sabas Carlos Vieira, Leonardo Halley Carvalho Pimentel, José Carlos Castelo Branco Ribeiro, Argemiro Ferreira de Andrade, Jerúsia Oliveira Ibiapina de Santana

**Keywords:** Meigs’ syndrome, CA 125 Antigen, Ovarian tumor, Ovarian carcinoma, Thecoma, Síndrome de Meigs, Câncer de ovário, Tumor ovariano, Carcinoma ovariano, Tecoma

## Abstract

**CONTEXT:**

Meigs’ syndrome consists of a benign ovarian tumor accompanied by ascites and hydrothorax. Elevated serum CA 125 levels in postmenopausal women with solid adnexal masses, ascites and pleural effusion are highly suggestive for malignant ovarian tumor. However, patients with Meigs’ syndrome can also have elevated serum CA 125 levels. The authors report a case of Meigs’ syndrome with elevated CA 125 level.

**OBJECTIVE:**

This is a case report of Meigs’ syndrome with elevated CA 125 level.

**CASE REPORT:**

A 65-year-old Brazilian woman had presented progressive dyspnea, weight loss and decline in general condition over the 7 months preceding admission to our service. In another hospital, the patient had been submitted to thoracic drainage due to pleural effusion. With recurrence of the pleural effusion and increase in abdominal volume due to ascites and a pelvic mass, the patient sought our service. Transvaginal ultrasound showed an extensive adnexal solid mass of 16.4 x 10.8 cm located in the pelvis without exact limits, and the serum CA 125 level was elevated. With a preoperative diagnosis of ovarian carcinoma, the patient was submitted to exploratory laparotomy, which revealed a left ovarian tumor. The frozen section diagnosis was thecoma. Total abdominal hysterectomy with bilateral salpingo-oophorectomy was performed. The histology of the specimen confirmed the diagnosis of thecoma. The patient was asymptomatic with a normal serum CA 125 level 20 months after the operation.

## RESUMO

**CONTEXTO:**

A síndrome de Meigs consiste de um tumor ovariano benigno em associação com ascite e hidrotórax. Níveis elevados de CA 125 sérico em mulheres pós-menopáusicas com massa anexial sólida, ascite e derrame pleural são muito sugestivos de tumor ovariano maligno. Entretanto, pacientes com síndrome de Meigs podem apresentar níveis elevados de CA 125. Os autores relatam um caso de síndrome de Meigs com CA 125 elevado.

**OBJETIVO:**

Relatar um caso de síndrome de Meigs com CA 125 elevado.

**RELATO DO CASO:**

Mulher de 65 anos, brasileira, apresentou dispnéia progressiva, perda de peso e queda do estado geral durante os últimos sete meses antes da admissão em nosso serviço. A paciente foi submetida, em outro hospital, a drenagem torácica devido derrame pleural. Com a recidiva do derrame pleural e aumento do volume abdominal por ascite e massa pélvica, a paciente procurou nosso serviço. O ultra-som transvaginal mostrou uma massa anexial sólida de 16,4 x 10,8 cm de limites imprecisos localizada na pélvis e o CA 125 sérico estava elevado. Com diagnóstico pré-operatório de carcinoma ovariano, a paciente foi submetida a laparotomia exploradora, que revelou um tumor ovariano esquerdo. Biópsia de congelação diagnosticou tecoma. Realizou-se uma histerectomia total abdominal com salpingo-ooforectomia bilateral. O exame histopatológico confirmou o diagnóstico de tecoma. A paciente encontrava-se assintomática e o CA 125 estava normal 20 meses após a operação.

## INTRODUCTION

Meigs’ syndrome consists of a benign ovarian tumor accompanied by ascites and hydro-thorax. Elevated serum carbohydrate antigen 125 (CA 125) levels in postmenopausal women with solid adnexal masses, ascites and pleural effusion are highly suggestive for malignant ovarian tumor. However, patients with Meigs’ syndrome can also have elevated serum CA 125 (a tumor marker) levels.^1,2^ We present a case of Meigs’ syndrome due to left ovarian thecoma with elevated CA 125.

## CASE REPORT

A 65-year-old Brazilian woman had presented progressive dyspnea, weight loss and decline in general condition over the 7 months preceding admission to our service. In another hospital, the patient had been submitted to thoracic drainage due to pleural effusion in her left lung and at that time she had begun therapeutic tests for tuberculosis, using isoniazid, rifampicin and pyrazinamide, without clinical improvement. With recurrence of the pleural effusion and increase in abdominal volume due to ascites and a pelvic mass, the patient sought our service.

Chest examination revealed dullness to percussion and lack of breath sounds in her left lung. There was arching in the lower abdomen due to a firm and immobile pelvic mass. The gynecological examination brought out evidence of arching of the vesicouterine and rectouterine pouches due to extrinsic compression, and the uterine cervix was normal. The patient was interned and submitted to pleural drainage and biopsy that indicated non-specific chronic pleurisy. Ultrasound showed an extensive adnexal solid mass of about 16.4 x 10.8 cm located in the pelvis, without exact limits. The serum CA 125 level was 319 IU/ml (normal is 5-35 IU/ml).

**Figure 1:**
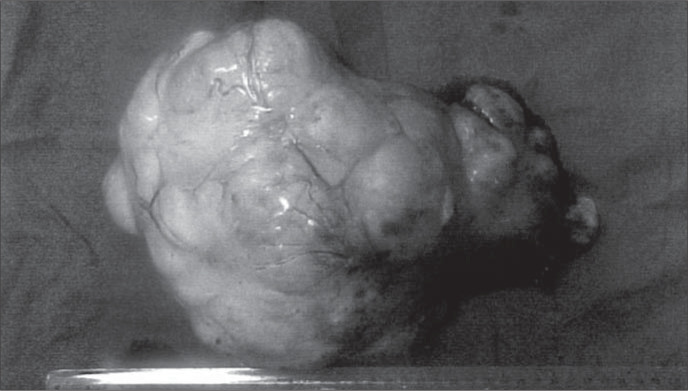
Macroscopic appearance of surgical specimen of a left ovarian thecoma.

**Figure 2:**
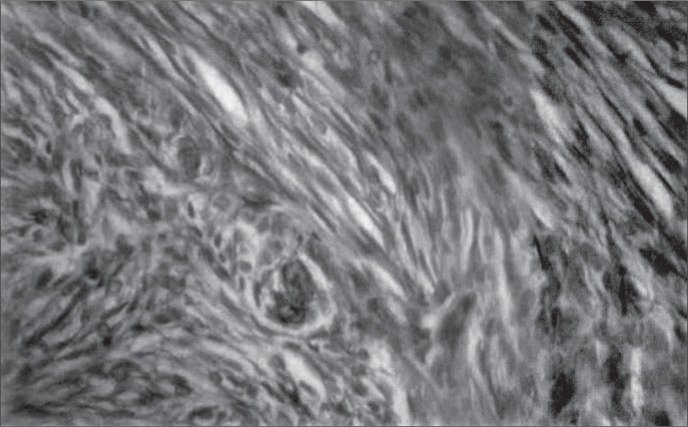
Microscopic appearance of left ovarian thecoma (hematoxylin-eosin, 400 x).

Due to clinical suspicion of malignant ovarian tumor, the patient was submitted to exploratory laparotomy that brought out evidence of the presence of serohematic ascites and a left ovarian tumor measuring 14 x 12 x 8 cm with a solid lobular appearance (Figure 1). The frozen section diagnosis of the ovarian tumor was thecoma. Total abdominal hysterectomy was performed, with bilateral salpingo-oophorectomy and cholecystectomy because of the presence of cholelithiasis. Histopathological examination of the tumor revealed a benign stromal lesion with tumor cells arranged in a fascicular fashion consistent with thecoma (Figure 2).

The postoperative period was uneventful and the patient was discharged from hospital on the 7^th^ postoperative day. The serum CA 125 level on the 4^th^ postoperative day was 406.3 IU/ml and was 53.7 IU/ml on the 30^th^ postoperative day. The patient was asymptomatic with a normal serum CA 125 level 20 months after the operation.

## DISCUSSION

Only 14 cases of Meigs’ syndrome with elevated CA 125 levels have been described in the literature.^1^ The most common histological types reported have been cellular fibroma (4 cases), fibroma thecoma (3 cases), fibroma (3 cases), thecoma (3 cases) and granulosa cell tumor (1 case). Serum CA 125 levels have ranged from 42.3 to 2,120 IU/ml, and one patient presented levels higher than 5,000 IU/ml.^1^

Thecoma is a rare ovarian tumor and it is associated with Meigs’ syndrome in just 2% of the cases.^2^ Several hypotheses might explain the formation of the ascitic fluid. The main theory is that the transudation mechanism through the tumor surface exceeds the capacity for peritoneal reabsorption. Another mechanism implicates the congestion of the peritoneal lymphatic vessels and regional veins caused by the tumoral mass itself or vasoactive substances released by the tumor.^1^ It is thought that the occurrence of hydrothorax is secondary to the passage of ascitic fluid to the pleural space through the diaphragm or diaphragmatic lymphatic vessels, or alternatively because of congenital defects, which are more common on the right side.^3^

The CA 125 tumor marker is generally elevated in patients with malignant ovarian tumor. It can, however, be elevated in benign disorders, such as endometriosis, pelvic inflammatory disease and uterine leiomyoma. Serum CA 125 levels can also increase in pericardial, pleural and peritoneal irritation or inflammation.^1.4^ Laparotomy and histopathological examination are required for the correct diagnosis and treatment of ovarian tumors, since elevated serum CA 125 levels can be falsely positive for ovarian malignancy.^1^

Postmenopausal women with clinical conditions of palpable pelvic masses, ascites, pleural effusion and elevated serum CA 125 levels probably have malignant ovarian tumors. However, thecoma forming part of Meigs’ syndrome is also a diagnostic possibility.

## References

[1] Abad A, Cazorla E, Ruiz F (1999). Meigs’ syndrome with elevated CA 125: case report and review of the literature. Eur J Obstet Gynecol Reprod Biol.

[2] Turan YH, Demirel LC, Ortac F (1993). Elevated CA 125 in Meigs syndrome. Int J Gynaecol Obstet.

[3] Agranoff D, May D, Jameson C, Knowles GK (1998). Pleural effusion and a pelvic mass. Postgrad Med J.

[4] Niloff JM, Knapp RC, Schaetzl E, Reynolds C, Bast RC (1984). CA125 antigen levels in obstetric and gynecologic patients. Obstet Gynecol.

